# DCS-ELM: a novel method for extreme learning machine for regression problems and a new approach for the SFRSCC

**DOI:** 10.7717/peerj-cs.411

**Published:** 2021-03-12

**Authors:** Osman Altay, Mustafa Ulas, Kursat Esat Alyamac

**Affiliations:** Firat University, Elazig, Turkey

**Keywords:** Extreme learning machine, Discrete-time chaotic systems, Chaotic maps, Regression algorithm, SFRSCC

## Abstract

Extreme learning machine (ELM) algorithm is widely used in regression and classification problems due to its advantages such as speed and high-performance rate. Different artificial intelligence-based optimization methods and chaotic systems have been proposed for the development of the ELM. However, a generalized solution method and success rate at the desired level could not be obtained. In this study, a new method is proposed as a result of developing the ELM algorithm used in regression problems with discrete-time chaotic systems. ELM algorithm has been improved by testing five different chaotic maps (Chebyshev, iterative, logistic, piecewise, tent) from chaotic systems. The proposed discrete-time chaotic systems based ELM (DCS-ELM) algorithm has been tested in steel fiber reinforced self-compacting concrete data sets and public four different datasets, and a result of its performance compared with the basic ELM algorithm, linear regression, support vector regression, kernel ELM algorithm and weighted ELM algorithm. It has been observed that it gives a better performance than other algorithms.

## Introduction

Feed-forward neural networks have been widely used since they were proposed (Rumelhart, Hinton & Williams, 1986). Traditional feed-forward neural networks generally use the first-order gradient method to optimize parameters. Feed-forward neural networks suffer from problems such as low convergence and local minimums (Huang et al., 2015). To deal with this problem, researchers have proposed different methods. These include feed-forward artificial neural network models developed with optimization methods such as artificial bee colony (Karaboga, Akay & Ozturk, 2007), hybrid particle swarm optimization (Al-kazemi & Mohan, 2002), differential evolution (Ilonen, Kamarainen & Lampinen, 2003) and genetic algorithm (Montana & Davis, 1989) during training. However, these methods still cannot provide the global optimal solution and need to be improved.

Lack of fast learning algorithms in artificial neural networks, training of artificial neural networks using traditional methods took hours and even days caused the need for a new method. As a result, the extreme learning machine (ELM) algorithm has emerged and ELM algorithm has been proposed by Huang, Zhu & Siew (2006). ELM is used to train single-layer feed-forward neural networks (SLFNs). It has been shown in various articles that the ELM algorithm provides a better global optimal solution when compared to traditional feed-forward neural networks. Theoretical studies have shown that even with randomly generated hidden nodes, ELM retains universal convergence ability over SLFNs.

Different versions of the ELM algorithm developed with different optimization methods and chaotic systems have been proposed in order to give a better global optimum solution. In 2005, Zhu et al. (2005) proposed the Evolutionary ELM algorithm using the differential evolutionary algorithm method. An ELM algorithm using the particle swarm optimization method was proposed by Xu & Shu (2006). In addition to these, an ELM algorithm developed by using different evolutionary optimization algorithms has also been proposed (Zhu et al., 2005; Xu & Shu, 2006; Silva, Pacifico & Ludermir, 2011). In addition to artificial intelligence-based optimization algorithms, there is also an ELM algorithm developed using chaotic systems (Huang et al., 2017; Yang, Wang & Yuan, 2013). Chaotic systems have also been used to develop optimization methods used in the ELM algorithm. Examples of these are the chaotic salp swarm optimization method (Mohanty et al., 2020) and the ELM algorithm improved by the chaotic moth-flame optimization method (Wang et al., 2017).

In this study, assignment of weight values and bias values was based on a determination using chaotic maps, not randomly. In the basic ELM algorithm, weight and bias values are assigned randomly. The random selection of bias and weight values seems to be the biggest obstacle to achieving the desired global optimum solution as a result of insufficient dispersion of the distributions. This causes repetition and generation of the same values when high values are needed due to the irregular operation of the random command (Yang, Wang & Yuan, 2013; Mohanty et al., 2020; Wang et al., 2017).

Chaotic system classes can be listed as discrete time, continuous time, time delay and hyper-chaotic systems. Each of these chaotic classes of systems has its own advantages and disadvantages. Discrete-time chaotic systems are used to determine the weight and bias values. Discrete-time chaotic systems have a significant advantage over other chaotic system models due to their high performance in computer applications with their simple mathematical models.

It is aimed to find the best bias and weight parameters by using discrete-time chaotic systems. It was observed that the proposed algorithm in the study achieved better results when compared with the basic ELM algorithm, linear regression (LR), support vector regression (SVR), kernel ELM (KELM) and weighted ELM (WELM). In particular, the proposed algorithm has found a better and generalized solution in data sets where the number of hidden neurons increases and long training period. A discrete-time chaotic systems-based extreme learning machine (DCS-ELM) algorithm has been proposed using discrete-time chaotic systems to improve the performance of the extreme learning machine algorithm. In the proposed algorithm, Chebyshev, iterative, logistic, piecewise and tent map discrete-time chaotic systems are used. The proposed DCS-ELM algorithm has been tested in 8 different data sets and it has been found to give better results in most of them.

## Extreme learning machine

Feed-forward neural networks are widely used in many different areas due to their capabilities. The first is to predict nonlinear mapping methods using direct input samples. The second is; It can offer models for natural and artificial classes. Lack of fast learning algorithms in artificial neural networks, training of artificial neural networks using traditional methods took hours and even days caused the need for a new method. As a result, the ELM algorithm has emerged (Huang, Zhu & Siew, 2006).

Traditionally, all parameters of feed forward networks have to be set (Gurgenc et al., 2020). For this reason, there is a dependency relationship between bias and weight values between different layers. Gradient descent based methods are mainly used in various learning algorithms of feed forward neural networks. Gradient descent based methods are sometimes very slow or can easily approach the local minimum. Too many iterations may be required to achieve better learning. Feed forward networks can be thought of as a linear model after the input weights and hidden layer trends are randomly selected. Output weights of feedforward networks can be determined analytically by simple generalized inverse study of hidden layer output matrices. The exit logic of ELM is also based on this situation and it has been shown in different data sets that it is a much faster and generalized model compared to traditional artificial neural networks (Huang, Zhu & Siew, 2006).

## Gradient-based solution

The gradient-based solution has traditionally been used to train single-hidden layer feed forward neural networks. Specifically, it is used to find the values of }{}{{\tilde w}_{i}},{{\tilde b}_{i}}, {\tilde{\rm \beta },}\left( {{i} = 1, \ldots ,{\tilde N}} \right) (Huang, Zhu & Siew, 2006) and its shown in Eq. (1).

(1) \Vert{H}( {{{{\tilde w}}_1}, \ldots ,{{{\tilde w}}_{{\tilde N}}},{{{\tilde b}}_1}, \ldots ,{{{\tilde b}}_{N}}}){\rm \beta } - {T}\Vert = \min {{w}_{i}}{{b}_{i}}{\rm \beta} \Vert{H}\left( {{{{\tilde w}}_1}, \ldots ,{{{\tilde w}}_{{\tilde N}}},{{{\tilde b}}_1}, \ldots ,{{{\tilde b}}_{N}}} \right){\rm \beta } - {T}\Vert

This corresponds to the minimum value of the cost function (Eq. (2));

(2) }{}{E} = \mathop \sum \limits_{{j} = 1}^{N} \mathop \sum \limits_{{i} = 1}^{{\tilde N}} {\left( {{{\rm \beta }_{i}}{g}\left( {{{w}_{i}} \times {{x}_{j}} + {{b}_{i}}} \right) - {{t}_{j}}} \right)^2}

If the }{}{H} value is unknown in the gradient-based learning algorithm, the algorithm usually starts looking for the minimum value of }{}{\rm H{\rm \beta} } - {T}. In the gradient-based minimization process, the weights \left( {{{w}_{i}},{{\rm \beta }_{i}}} \right) and the bias value are expressed as }{}{{b}_{i}}. }{}{W} parameter is iteratively adjusted as Eq. (3) (Huang, Zhu & Siew, 2006).

(3) }{}{{W}_{k}} = {{W}_{{k} - 1}} - {n}\displaystyle{{\partial {E}\left( {W} \right)} \over {\partial {W}}}

Here }{}{ n} is learning rate. The learning algorithm popularly used in feedforward neural networks is a back propagation learning algorithm that can be efficiently calculated by the propagation of gradients from output to input. There are several problems with the back propagation learning algorithm (Huang, Zhu & Siew, 2006; Ulas et al., 2019);

- When the learning rate }{}{ n} value is small, the learning algorithm converges very slowly. When the value of }{}{n} is large, the algorithm becomes unstable and diverges.
- One of the factors affecting the backpropagation learning algorithm is the presence of local minimums. It is not desired that the learning algorithm stop at the local minimum instead of the global minimum.
- Artificial neural network; He may have over-trained or poor generalization performance using the back propagation learning algorithm. Therefore, valid and appropriate stopping methods are required in the cost function reduction procedure.
- Gradient-based learning takes a lot of time in most applications.

In the ELM algorithm proposed to solve these problems in gradient-based algorithms, these problems have been eliminated and a more efficient learning algorithm is obtained for feed-forward neural networks (Huang, Zhu & Siew, 2006).

## Least squares norm

Unlike traditional function approximation theories that require adjusting input weights and hidden layer bias, input weights and hidden layer bias values can be assigned randomly only if the activation function is infinitely different. Contrary to the common understanding that all parameters of feedforward neural networks need to be tuned, the input weights and bias values in the hidden layer do not need to be adjusted, and the hidden layer output matrix }{}H can actually remain unchanged. The linear system is the analysis of }{}H{\rm \beta} = T with the least squares norm \hat {\rm \beta}. The solution for this is given in Eq. (4).

(4) }{}{H}\left( {{{w}_1}, \ldots ,{{w}_{{\check N}}},{{b}_1}, \ldots ,{{n}_{{\hat N}}}} \right){\hat {\rm \beta} } - {T} = \mathop {\min }\limits_{\rm {\beta} } {H}\left( {{{w}_1}, \ldots ,{{w}_{\check {N}}},{{b}_1}, \ldots ,{{n}_{{\hat N}}}} \right){\rm \beta } - {T}

If the }{}{\hat N} number of hidden nodes is equal to the }{}{N} number of samples, and the }{}{H} matrix is square and reversible, the input weight vectors }{}{{w}_{i}} and hidden bias values }{}{{b}_{i}} can be chosen randomly. However, in most real problems, the number of hidden nodes is much less than the number of different training instances. }{}{H} is a non-square matrix. There may be conditions that cannot be met at }{}{H{\rm{\beta}} } = {T}. The smallest norm leasts squares solution of linear system is given in the Eq. (5).

(5) }{}{\rm \beta } = {{H}^{*}}{T}

Here, the inverse of the }{}{H} matrix is taken using Moore–Penrose, }{}{{H}^{*}}.

In short, ELM, in a given training set \aleph = \left\{ {\left( {{{x}_{i}},{{t}_{i}}} \right)\parallel {{x}_{i}} \in {{R}^{n}},{{t}_{i}} \in {{R}^{m}},{i} = 1, \ldots ,{N}} \right\}, activation function }{}{g}\left( {x} \right) and hidden nodes }{}{\tilde N};

Step 1: Assign randomly; weight }{}{{w}_{i}} and bias value }{}{{b}_{i}}, }{}{i} = 1, \ldots ,{\tilde N}.

Step 2: Compute the hidden layer output matrix }{}{H}.

Step 3: Calculate the output weight }{}{\rm \beta } = {{H}^{*}}{T}, }{}{T} = {\left[ {{{t}_1}, \ldots ,{{t}_{N}}} \right]^{T}}. The inverse of the }{}{H} matrix is taken using Moore–Penrose }{}{{H}^{*}}.

**Figure 1:**
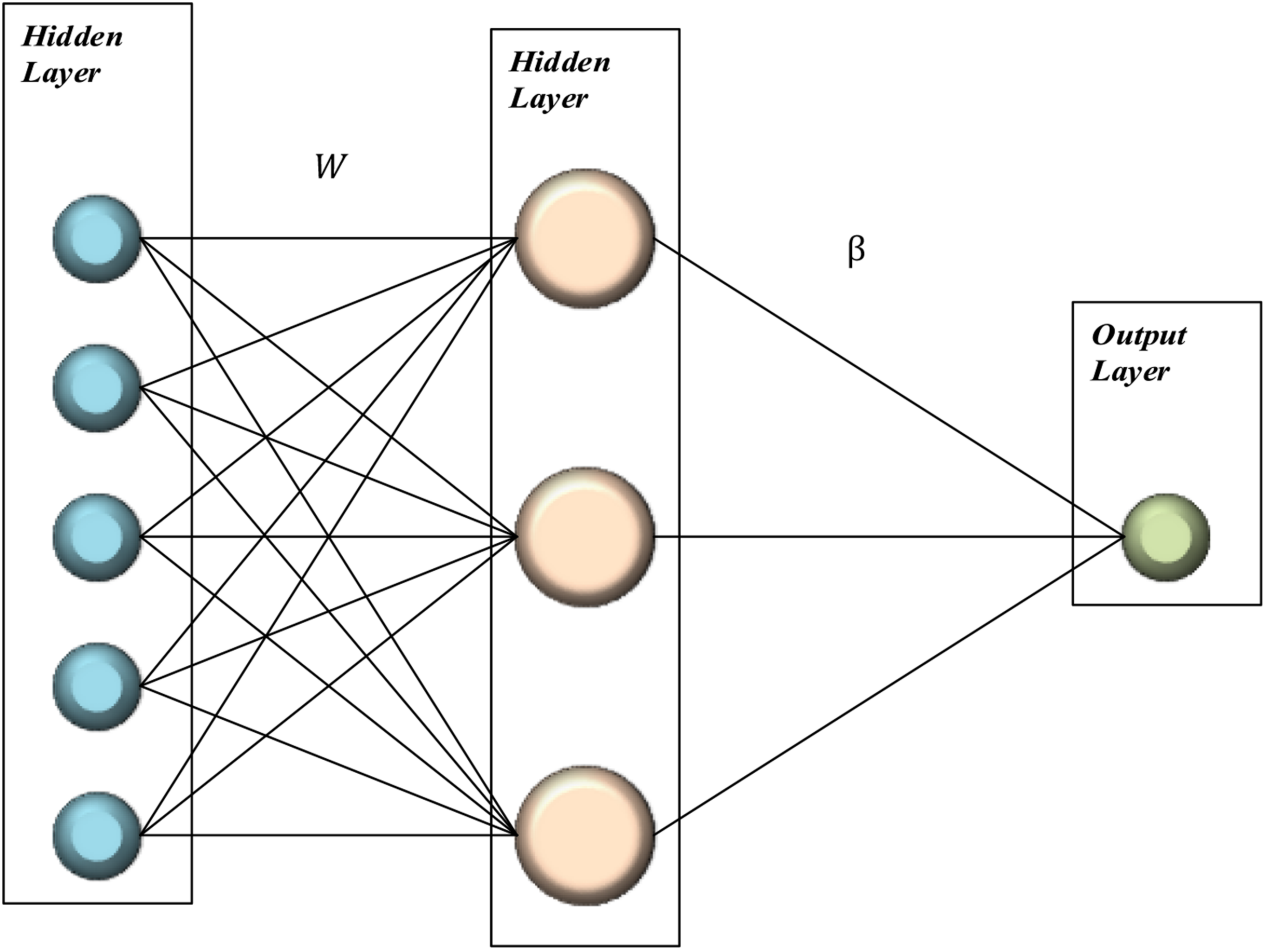
Basic representation of ELM.

In summary, in the ELM algorithm; It is randomly generated with the weight and bias values adjusted. Traditional feed forward neural networks train the network recursively, while in the ELM algorithm, the process is done analytically (Bilhan et al., 2018). n the ELM algorithm, Moore–Penrose generalized inversed has been used to eliminate the disadvantages of recursive learning algorithms (Altay & Ulas, 2019). In this way, a nonlinear system has been transformed into a linear system (Huang, Zhu & Siew, 2006; Huang et al., 2011). The basic representation of the ELM algorithm is given in Fig. 1.

### Activation function

Different activation functions are used in ELM as in artificial neural networks. There is no input information about which activation function will be used according to the problem. Activation functions are completely determined by trial and error methods. Hard limit, sine and sigmoid activation functions were used in the DCS-ELM algorithm suggested in the study. Hard limit activation function is shown in Eq. (6) (Huang et al., 2011).

(6) }{}{G}\left( {{a},{b},{x}} \right) = \left\{ {\matrix{ {1,{\rm if}\; a \times x - b \ge 0\; } \cr {0,\; \; \; \; \; \; {\rm otherwise}\; \; \; \; \; \; \; } \cr } } \right.

### Chaos theorem

Chaos has been in every event since the existence of the world. Chaos basically has a certain stable and unique structure. Chaotic systems are able to be stable as long as they can withstand different disturbing effects from the outside of their own disorder (Baykal & Beyan, 2004). There are differences between chaotic systems and random systems. Although chaos and random systems are perceived as the same by many, there is a very basic and distinctive difference between them. This difference is that chaotic systems have an order in disorder. After the concept of chaos emerged, people working in this field regarded order as spontaneous systems in chaotic systems and observed that irregular behavior was a creative process (Baykal & Beyan, 2004).

Chaotic systems can be defined as systems with unpredictable and random behavior with the shortest definition. The most basic feature of chaos is that it depends on the initial conditions. In a chaotic system, although the initial conditions are very close to each other, its orbits have no relation with each other and the orbits diverge from each other. There is very little difference between very close values that occur in initial conditions and this difference can be considered as measurement error. In contrast, chaotic systems increase exponentially and the state of the system becomes indeterminable after a short time. Chaotic systems are deterministic, contrary to popular belief, and should not be confused with stochastic systems. In a system, chaos is not a random external effect, but the internal dynamics of the system itself (Baykal & Beyan, 2004; Ozer, 2010).

In order for a systemic behavior to be called chaotic, it must comply with the following conditions.

- It must be sensitive to the starting conditions, that is to say excessively dependent,
- It must contain a nonlinear element,
- Discrete-time systems should have at least a first order, continuous time systems should have at least a third order differential equation.

**Figure 2:**
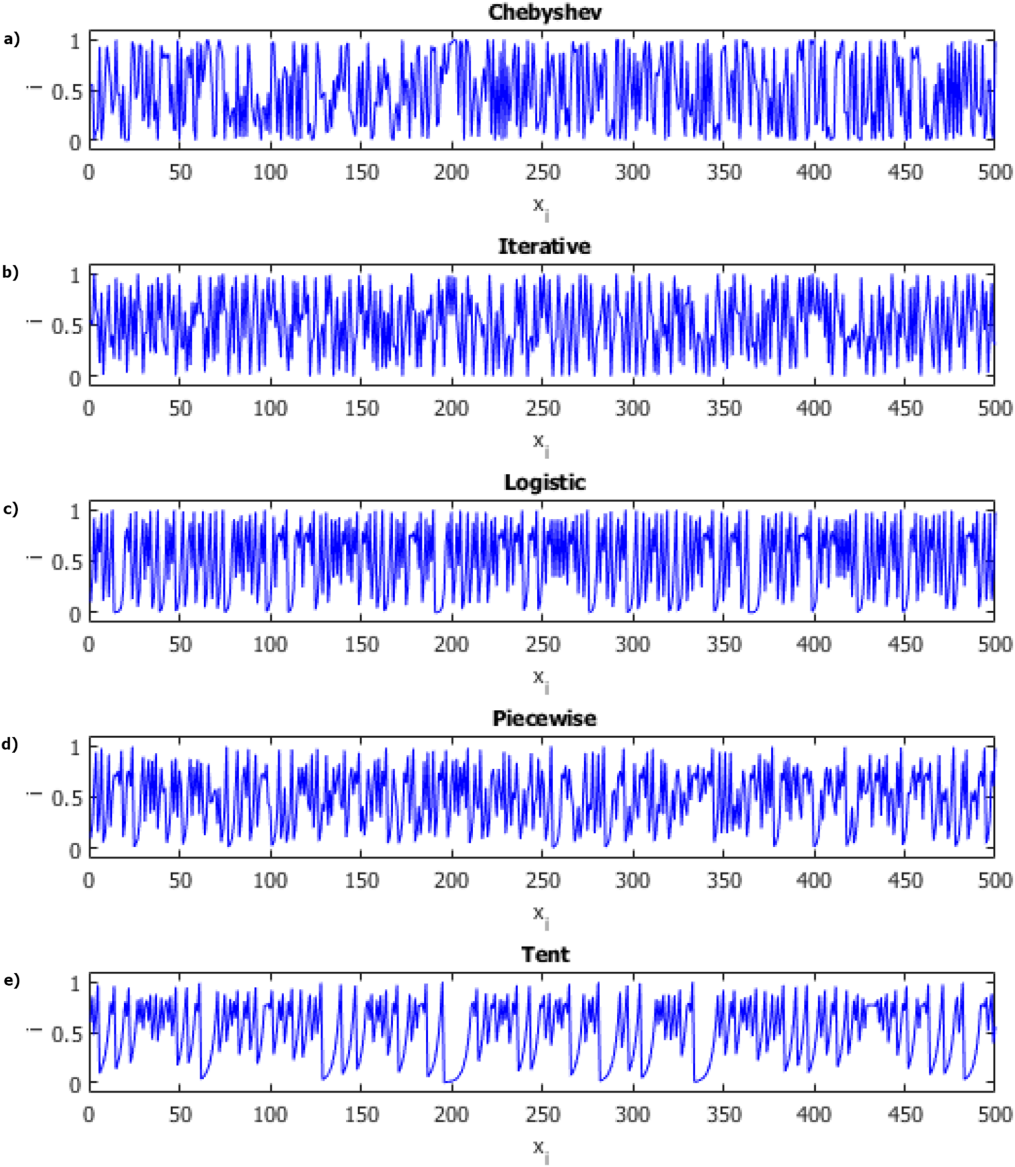
Distributions of chaotic maps. (A) Chebyshev. (B) Iterative. (C) Logistic. (D) Piecewise. (E) Tent.

Chaos theory has a much broader structure than that summarized here. There are many derivatives of chaotic systems. These chaotic system classes; It can be listed as discrete time, continuous time, time delay and hyper chaotic systems. Each of these chaotic classes of systems has its own advantages and disadvantages. Discrete-time chaotic systems have a significant advantage over other chaotic system models due to their high performance in computer applications with their simple mathematical models. Because of these advantages, we focused on discrete-time chaotic systems. Chaotic maps and their equations used in this study are listed in Table 1 and Fig. 2 includes sample distribution charts of chaotic maps.

**Table 1:** Equations and parameters of chaotic maps.

Chaotic Maps	Equations	Parameters
Chebyshev Map	}{}{X_{n + 1}} = {\rm cos}\left( {kco{s^{ - 1}}{x_n}} \right)	–
Iterative Map	}{}{X_{n + 1}} = sin\left( {\displaystyle{{a{\rm \pi} } \over {{x_n}}}} \right)	}{}a = 0.9
Logistic Map	}{}{X_{n + 1}} = a{X_n}\left( {1 - {X_n}} \right)	}{}a = 4
Piecewise Map	}{}{x_{n + 1}} = \left\{ {\matrix{ {\matrix{ {\displaystyle{{{x_n}} \over P},\; \; 0 \le {x_n} \lt P} \cr {\displaystyle{{{x_n} - P} \over {0.5 - P}},\; \; P \le {x_n} \lt 0.5} \cr } } \cr {\displaystyle{{1 - P - {x_n}} \over {0.5 - P}},\; \; 0.5 \le {x_n} \lt1 - P} \cr {\displaystyle{{1 - {x_n}} \over P},\; \; \; \; \; \; \; \; \; \; \; \; \; 1 - P \le {x_n} < 1} \cr } } \right.	}{}P = 0.4
Tent Map	}{}X_{n+1} = \left\{ \matrix{\displaystyle{{{x_n}} \over 0.7},\; \; {x_n} \leq 0.7 \cr \displaystyle\frac {{10}\over{3}} X_n(1-X_n), de \check gilse \right.	–

### Proposed DCS-ELM

Recently, chaotic number sequences replacing random number sequences have been used in secure communication (Caponetto et al., 1998), improving the performance of optimization methods (Alatas, 2010; Altay & Alatas, 2020), artificial neural networks (Nozawa, 1992) and nonlinear circuits (Arena et al., 2000). More successful results have been obtained in some applications.

The parts to be determined by the user in the basic ELM algorithm are determined as the activation function and the number of hidden layers. ELM algorithm randomly generates input weights and bias value. As a result of the random generation of these values, the distribution of the values is not good, and the desired performance cannot be obtained from the ELM algorithm from time to time. The basic ELM algorithm is shown in Table 2.

**Table 2:** Basic ELM algorithm.

ELM
In a given training set \aleph = \left\{ {\left( {{x_i},{t_i}} \right)\parallel {x_i} \in {R^n},{t_i} \in {R^m},i = 1, \ldots ,N} \right\}, activation function }{}g\left( x \right) and hidden nodes \tilde N; ***Step 1:*** Assign randomly; weight }{}{w_i} and bias value }{}{b_i}, \; i = 1, \ldots ,\tilde N. ***Step 2:*** Compute the hidden layer output matrix \bf{H}. ***Step 3:*** Calculate the output weight }{}{\rm \beta} = {\bi{H}^\bi{*}}\bi{T}, \bi{T} = {\left[ {{\bi{t}_1}, \ldots ,{\bi{t}_\bi{N}}} \right]^\bi{T}}. The inverse of the\bi{\; H} matrix is taken using Moore–Penrose }{}{\boldsymbol{H}^\bf{*}}.

In the proposed algorithm, input weights and bias values are created by using chaotic maps instead of random. In this way, it is aimed to eliminate the disadvantages caused by random generation. The flow of the proposed DCS-ELM is given in Table 3. Performance of the proposed algorithm according to ELM and basic machine learning algorithm is shown in the next sections.

**Table 3:** DCS-ELM algorithm.

DCS-ELM
In a given training set \aleph = \left\{ {\left( {{x_i},{t_i}} \right)\parallel {x_i} \in {R^n},{t_i} \in {R^m},i = 1, \ldots ,N} \right\}, activation function }{}g\left( x \right) and hidden nodes \tilde N; ***Step 1:*** Assign using chaotic maps; weight }{}{w_i} and bias value }{}{b_i}, }{}i = 1, \ldots ,\tilde N. ***Step 2:*** Compute the hidden layer output matrix \bi{H}. ***Step 3:*** Calculate the output weight }{}{\rm \beta} = {\bi{H}^\bi{*}}\bi{T}, \bi{T} = {\left[ {{\bi{t}_1}, \ldots ,{\bi{t}_\bi{N}}} \right]^\bi{T}}. The inverse of the \bi{\; H} matrix is taken using Moore–Penrose }{}{\boldsymbol{H}^\bf{*}}.

### 10-k cross validation

In the 10-k cross validation method, the data set is primarily randomly distributed. Then the data set was divided into 10 parts. While each piece was used as a test data set, the remaining 9 pieces were used as a training set, respectively. With the 10-k cross validation method, more consistent results can be obtained by using each data in the data set as test data. A simple representation of the 10-k cross validation method is given in the Table 4.

**Table 4:** 10-k cross-validation simple representation.

Test	*Train*	*Train*	*Train*	*Train*	*Train*	*Train*	*Train*	*Train*	*Train*
*Train*	**Test**	*Train*	*Train*	*Train*	*Train*	*Train*	*Train*	*Train*	*Train*
*Train*	*Train*	**Test**	*Train*	*Train*	*Train*	*Train*	*Train*	*Train*	*Train*
*Train*	*Train*	*Train*	**Test**	*Train*	*Train*	*Train*	*Train*	*Train*	*Train*
*Train*	*Train*	*Train*	*Train*	**Test**	*Train*	*Train*	*Train*	*Train*	*Train*
*Train*	*Train*	*Train*	*Train*	*Train*	**Test**	*Train*	*Train*	*Train*	*Train*
*Train*	*Train*	*Train*	*Train*	*Train*	*Train*	**Test**	*Train*	*Train*	*Train*
*Train*	*Train*	*Train*	*Train*	*Train*	*Train*	*Train*	**Test**	*Train*	*Train*
*Train*	*Train*	*Train*	*Train*	*Train*	*Train*	*Train*	*Train*	**Test**	*Train*
*Train*	*Train*	*Train*	*Train*	*Train*	*Train*	*Train*	*Train*	*Train*	**Test**

### Evaluation metrics

In the study, using evaluation criteria are R-squared, root mean absolute error (RMSE) and mean absolute error (MAE). The equations of the evaluation criteria used are expressed as follows:

(7) }{}{{R}^2} = 1 - \left( {\displaystyle{{\mathop \sum \nolimits_{j} {{\left( {{{t}_{j}} - {{o}_{j}}} \right)}^2}} \over {\mathop \sum \nolimits_{j} {{\left( {{{t}_{j}} - {\hat t}} \right)}^2}}}} \right)

(8) }{}{\rm RMSE} = \sqrt {\left( {\displaystyle{1 \over {p}}} \right) \times \mathop \sum \limits_{j} {{\left| {{{t}_{j}} - {{o}_{j}}} \right|}^2}}

(9) }{}{\rm MAE} = \displaystyle{1 \over {p}}\mathop \sum \limits_{{i} = 1}^{p} \left| {{{t}_{i}} - {{o}_{j}}} \right|

where }{}{o} are the experimental values, }{}{ t} are the predicted values of the machine learning algorithms, \hat {\rm t} are the average of all experimental values and }{}{p} are the number of samples.

## Datasets

In this section, the data sets used to evaluate the performance of the proposed DCS-ELM algorithm and other algorithms are explained. First, SFRSCC data set is explained and then public data sets are explained.

### Self-compacting steel fiber concrete

In the application of the proposed algorithm, a special type of concrete, self-compacting steel fiber concrete is used. A total of 4 different concrete tests were selected from the fresh and hardened concrete tests. V-funnel, T50 and slump-flow tests used to determine fresh concrete performance and concrete compressive strength tests used to determine the performance of hardened concrete were used. In the selection of the data set, machine learning methods have not been applied before, they have the same number of input parameters, but the effect values of the parameters are different according to the experiments, there are not enough data sets in the literature and it is more difficult to obtain a successful performance with machine learning methods compared to different data sets. The data sets used were obtained from our own experiments and theses and articles obtained from the literature. 60 data sets were used in the models designed for V-funnel, 108 data sets in the model designed for T50, 122 data sets in the model designed for slump-flow, and 67 data in the model designed for compressive strength. The data in Table 5 are obtained from the experimental studies and the studies in Table 6 are obtained from the literature. The input parameters in the data set are cement (C), silica fume+silica powder+stone fume (S), fly ash (FA), maximum aggregate size (Dmax), fine aggregate (Fi), coarse aggregate (CA), water (W), chemical additive (A), amount of steel fiber (StF), diameter of steel fiber (FD) and length of steel fiber (FD). The output parameters in the data set are v-funnel (VF), T50, slump-flow (SF) and compressive strength (Fc). Silica fume, silica powder and stone fume are reduce the workability of the fresh concrete takes as group (Altay, Ulas & Alyamac, 2020). The effect of this group on the performance of concrete that has been hardened before 28 days is negligible.

**Table 5:** Data from experiment, input and output parameters of SFRSCC for v-funnel, T50, slump-flow and compressive strength models.

Mix Code	C kg/m^3^	S kg/m^3^	D_max_ (mm)	Fi kg/m^3^	CA kg/m^3^	W L	A L	StF kg/m^3^	FD mm	FL mm	VF (s)	T50 (s)	SF (cm)	f_c_ (MPa)
C-1	400	40	16	874	715	240	6	24	0.75	30	5.73	3.9	66	45.5
C-2	380	80	16	901	737	216	5.4	24	0.75	30	9.75	1.9	64	55.8
C-3	380	80	16	901	737	216	5.4	55	0.75	30	7.26	2.1	63	52.6
C-4	420	100	16	873	715	228	5.7	24	0.75	30	12.88	2.9	75	36.2
C-5	420	200	16	819	670	228	5.7	24	0.75	30	6.56	2	81	49.8
C-6	420	200	16	819	670	228	5.7	55	0.75	30	–	2.3	77	46.6
C-7	400	140	16	819	670	240	6	24	0.75	30	5.64	1.3	75	51.6
C-8	420	100	8	1399	0	285.6	6.3	24	0.75	30	–	–	–	36.3

**Table 6:** Data from literature, input and output parameters of SFRSCC for v-funnel, T50, slump-flow and compressive strength models.

Ref	C kg/m^3^	S kg/m^3^	FA kg/m^3^	D_max_ (mm)	Fi kg/m^3^	CA Kg/m^3^	W l	A kg/m^3^	StF kg/m^3^	FD mm	FL mm	VF s	T50 s	SF cm	f_c_ MPa
Korkmaz (2011)	350	0	150	16	865.3	787 793.8	200	12.5	40 60	0.75 0.9	30 60	13 15	3 3.8	54 66	49.2 54.1
Nis (2017)	420	0	180	10	777	636	120	3.6 4.2	39 78	0.16 0.55	13 35	6 6.5	3 4	79 80	58.9 62
Rao & Ravindra (2010)	390	0	210	16	735 749	769 783	186	2.9 3	39.3 117.8	0.92	13.8 32.2	7.4 11.8	4.4 5	56.5 68.5	45.2 47.6
Bozkurt (2009)	350	0	150	16	865	797 798	200	4.4	46 50	0.1 0.6	40 80	11 18	1.9 3.2	69 75	62.4 72.6
Kassimi (2013)	475	0	0	20	801 813	745 763	200	5.1	24 39	0.55	30	2.4 2.6	1.1 1.3	68 70	47.9 50.6
Tezel (2010)	350	561	150	16	494	816	164.5	4.2 5.25	10 90	0.16 0.9	13 60	13 60	4 10	71 74	–
Sahmaran & Yaman (2007)	250	70	250	19	889 924	530 549	205 226	11.8	60	0.16 0.55	6 30	2.7 2.8	2	66 70	–
Sahmaran, Yurtseven & Yaman (2005)	500	70	250	19	977	578	200	9.5	60	0.16 0.55	6 30	9.2	2.6 4.3	62 67.5	
Corinaldesi & Moriconi (2009)	500	58 70	0	16	1,080	420	200	7	50	0.7	30	5 7	1 3	66 70	–
Gencel et al. (2011)	400	0	120	16	427.7 431.7	1295.6 1307.7	160	6	15 60	0.5	30	16.4 20.7	4.3 4.7	58.2 69.2	–
Korkut et al. (2017)	350	30	100	16	870	750	140	5.6	19.6 58.8	0.75 0.9	30 60	6.1 18.1	1.9 5.1	59 68	–
Berbergil (2006)	350	0	150	16	738 744	749 760	235	4.5 8	30 60	0.75	60	4.6 12.3	–	65 71	49.2 54.1
Yıldırım, Sertbaş & Berbergil (2007)	350	0	150	16	731 736	747 752	235	3.15 5.6	30 60	0.75	60	5.09 12.3	–	–	49.2 55.6
Dinç (2007)	440 824	308 623	0	16	315	610	198	6.6 8.2	78	0.75	30	–	9 15	63 67	–
El-Dieb & Taha (2012)	350 500	40 42	0	19	687 736	1030 1104	173 176	7 10	40 160	0.16 0.5	8 30	–	8.5 17	52 68	–
Deeb (2013)	500	275	0	10	700	833	138	19	39	0.6	30	–	3	76	–
Ouedraogo (2018)	480	0	0	16	1,087	592	192	10.43 11.3	46.8	0.55 1	30 50	–	2.7 4.9	64 66	68.2 71.1
Pająk & Ponikiewski (2013)	490	0	0	8	808	808	201	19	40 120	0.4 0.8	12.5 30	–	2 6	64 68	80.1 98.2
Frazão et al. (2015)	413	353	0	19	908	640	127.8	7.8	60	0.5	30	–	15.6	66.7	–
Torrijos, Barragan & Zerbino (2008)	334	100	0	18	939	775	164	12.7	25	1	50	–	1.7	–	
Majdzadeh (2003)	420	24.7	49.4	10	695	1042	123.5	20	58.9 78.5	0.25 1	6.5 50	–	–	61 72	–
Jansson et al. (2012)	357 368	172 207	0	16	829 969	608 763	189 202	1.3	20 80	0.65	30	–	–	–	50 59
Long et al. (2014)	420 500	0	0	20	815	957	247 252	1.5 1.9	20	0.7	30 60	–	–	–	37.3 63.7

### Public datasets

The energy, house and servo data sets obtained from public data set sharing platform UCI are explained (Dua & Graff, 2019).

### Energy

Energy data set consists of 8 inputs and 2 outputs. Input values consist of relative compactness, surface area, wall area, roof area, overall height, orientation, glazing area and glazing area distrubution. Output values consist of heating load and cooling load. It has been examined separately for two different output values. There are 768 sample data in the data set created by considering the output variables of different buildings (Tsanas & Xifara, 2012).

### House

In the House data set, between June 2012 and May 2013, data from 4 different regions is beyond the supply and demand circle. There are a total of 414 sample data in the data set. The input parameters of the data set consist of 6 different parameters: transaction date, age, distance to the nearest MRT station, the number of convenience stores in the living circle on foot and the geographic coordinate (latitude and longitude). The exit value is the price of the house (Yeh & Hsu, 2018).

### Servo

There are 167 sample data in the servo data set. The input parameters of the data set are engine, screw, pgain and vgain. Output values constitute the rise time of the servomechanism. The dataset created by Karl Ulrich covers a nonlinear phenomenon (Quinlan, 1992, 1993).

## Results and discussion

In this study, the DCS-ELM algorithm, which was proposed for the first time using chaotic maps, was tested in 8 different regression datasets. In this section, first of all, the performances of DCS-ELM and other algorithms proposed on public data sets were examined and compared. Then, performances of DCS-ELM and other algorithms in SFRSCC datasets were examined and compared. Finally, a general evaluation of DCS-ELM and other algorithms proposed on 8 different data sets was made according to the RMSE value.

### Performance experiment results on public data sets

The proposed DCS-ELM algorithm using 5 different chaotic maps on 4 different public data sets is compared with the, LR (Altay et al., 2020), SVR (Altay et al., 2020), WELM (Ulas et al., 2019), KELM (Ulas et al., 2019; Yang et al., 2018) and basic ELM algorithm (Cao et al., 2018). LR and SVR algorithms are used with basic property parameters. The number of input, output, activation function and hidden neuron used in DCS-ELM, basic ELM, WELM and KELM are given in the Tables 7 and 8. The 10-k cross validation method was used to test the designed models. The basic ELM, WELM and KELM algorithms were run 100 times and the R^2^, RMSE and MAE values were averaged. Table 9 shows the results of basic ELM, LR, SVR, WELM, KELM and DCS-ELM algorithms for public data sets.

**Table 7:** Architecture of ELM, DCS-ELM and WELM.

	Hidden neuron	Activation function	Output neuron	Input neuron
Energy1	200	Sine	1	768
Energy2	300	Sine	1	768
House	20	Hardlim	1	414
Servo	50	Sigmoid	1	167

**Table 8:** Architecture of KELM.

	Kernel parameter	Activation function	Output neuron	Input neuron
Energy1	6	RBF kernel	1	768
Energy2	6	RBF kernel	1	768
House	6	RBF kernel	1	414
Servo	6	RBF kernel	1	167

**Table 9:** Results of public datasets.

	EM	LR	SVR	WELM *k* = 100	KELM *k* = 100	ELM *k* = 100	DCS-ELM Chebyshev	DCS-ELM Iterative	DCS-ELM Logistic	DCS-ELM Piecewise	DCS-ELM Tent
Energy1	R^2^	0.9200	0.9100	0.9336	0.9055	0.9809	0.8672	0.9905	0.8723	0.9826	0.9794
	RMSE	2.9421	2.0973	2.5596	3.0598	1.5470	5.5700	1.2450	4.9494	1.6421	1.2402
	MAE	2.0877	2.0435	1.9333	2.3510	1.0524	3.1354	0.8039	2.8124	1.0862	0.8969
Energy2	R^2^	0.8900	0.8800	0.9516	0.8838	0.9730	0.9478	0.9779	0.9626	0.9708	0.9636
	RMSE	3.2188	3.2484	2.0968	3.1851	2.0885	2.2023	1.7219	2.4084	1.9607	2.6307
	MAE	2.2643	2.2441	1.5415	2.3735	1.1709	1.5281	1.0744	1.5189	1.1168	1.2881
House	R^2^	0.5700	0.5600	0.6041	0.0000	0.5677	0.5932	0.6087	0.5676	0.6143	0.5292
	RMSE	8.9474	9.041	8.0768	29.2569	7.3118	7.2347	6.6873	7.4086	6.5335	7.2982
	MAE	6.2745	6.2238	5.7113	24.6785	5.5837	5.7505	5.0731	5.7930	5.2752	5.7343
Servo	R^2^	0.4800	0.1700	0.4936	0.6997	0.7893	0.7189	0.8271	0.2270	0.8265	0.7924
	RMSE	1.1348	1.4304	1.0887	0.7698	0.4511	0.5118	0.3574	0.8743	0.6183	0.3832
	MAE	0.9169	0.7850	0.7978	0.5153	0.3549	0.4323	0.2626	0.6280	0.4905	0.2798

### A new approach for the SFRSCC using DCS-ELM

SFRCSCC’s fresh and hardened concrete experiments performances were predicted using the proposed DCS-ELM algorithm using the basic ELM algorithm and 5 different chaotic maps. Parameters used in ELM and DCS-ELMs are taken exactly the same in all designed models in order to ensure a healthy comparison. The input, output, activation function and the number of hidden neurons of the basic ELM algorithm, WELM and DCS-ELM are shown in Table 10. KELM algorithm architecture shown in Table 11. In order to compare the ELM algorithm with the chaotic map-based ELM algorithms, the ELM algorithm was run 100 times and the evaluation criteria were averaged. All designed models were tested using the 10-k cross validation test method. R^2^, RMSE and MAE values were calculated separately for each model.

**Table 10:** Parameters of the ELM, WELM and DCS-ELM for SFRSCC.

	Hidden Neuron	Activation function	Output Neuron	Input Neuron
Vfunnel	5,000	Hard Limit	1	11
T50	5,000	Hard Limit	1	11
Slump-flow	5,000	Hard Limit	1	11
fc	5,000	Hard Limit	1	11

**Table 11:** Parameters of the KELM for SFRSCC.

	Kernel parameter	Activation function	Output Neuron	Input Neuron
Vfunnel	6	Lin kernel	1	11
T50	6	Lin kernel	1	11
Slump-flow	6	Lin kernel	1	11
fc	6	Lin kernel	1	11

**Figure 3:**
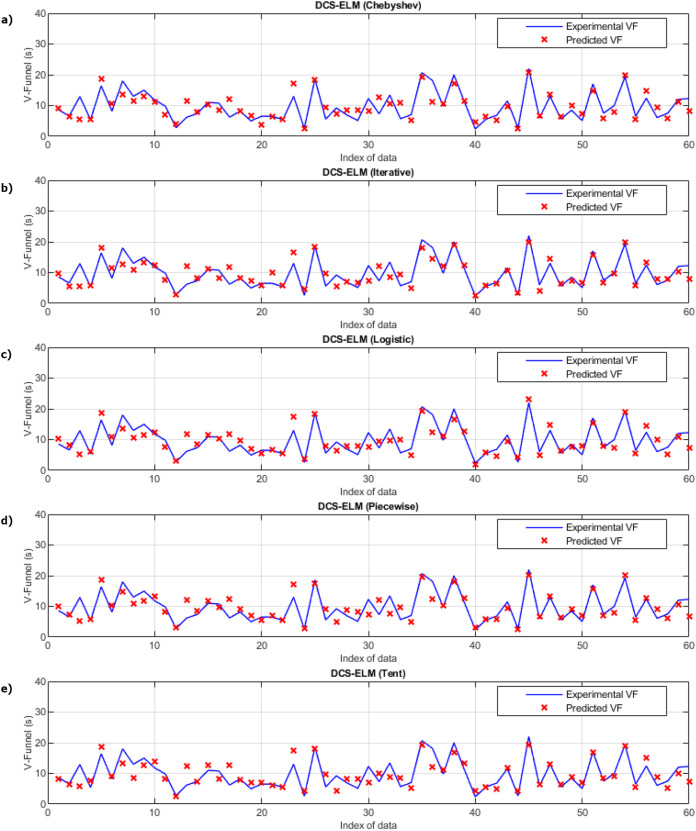
Experimental and predictive values of DCS-ELM algorithm in the V-funnel experiment. (A) Chebyshev. (B) Itaretive. (C) Logistic. (D) Piecewise. (E) Tent.

**Figure 4:**
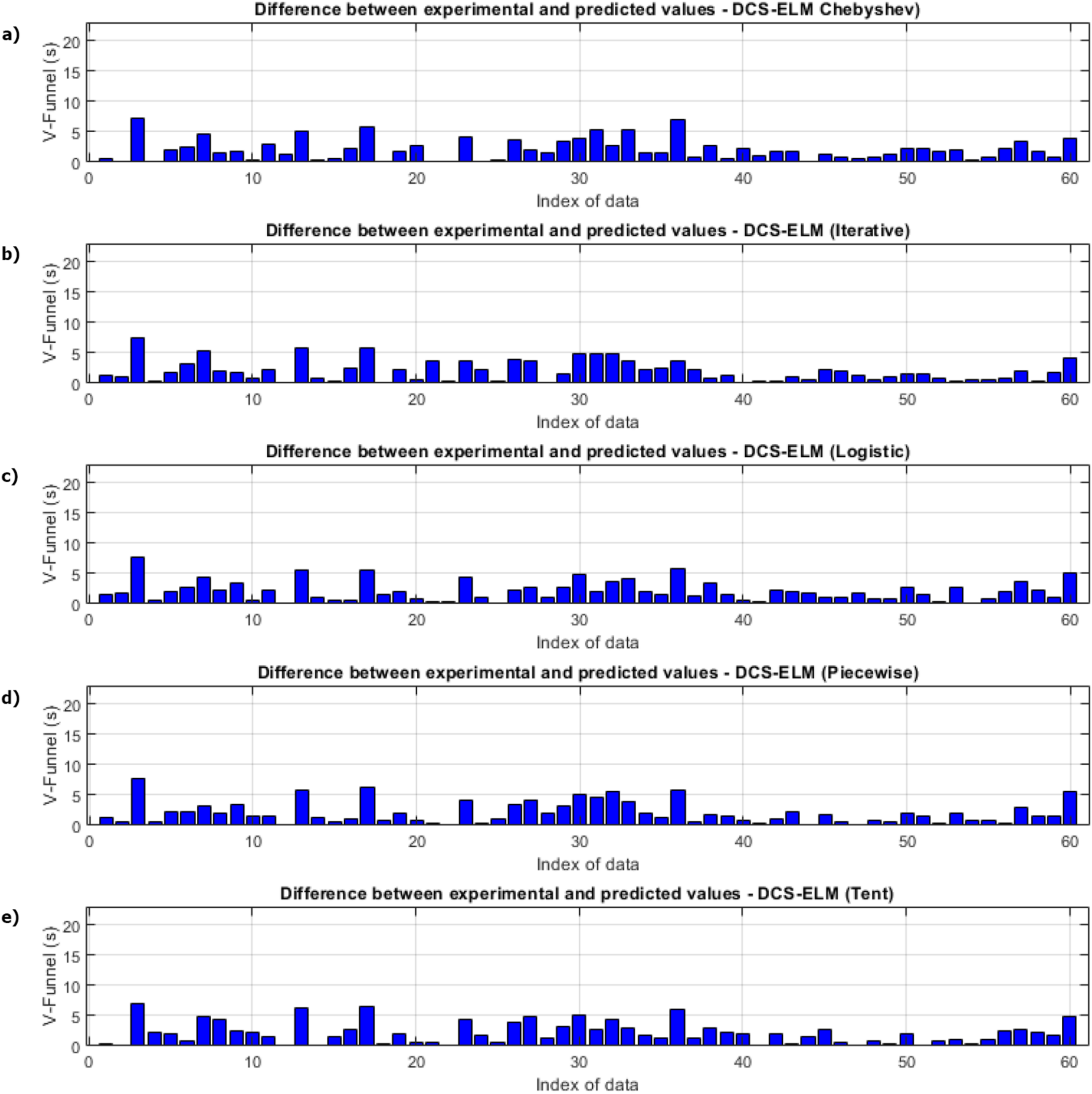
Differences between the experimental and predictive values of DCS-ELM algorithm in the V-funnel experiment. (A) Chebyshev. (B) Iterative. (C) Logistic. (D) Piecewise. (E) Tent.

In Fig. 3, the ELM algorithm of the v-funnel experiment and the prediction and experimental values of 5 different DCS-ELM algorithms are given, and Fig. 4 shows the differences between the prediction and experimental values. As it can be understood from Fig. 3 and Fig. 4, ELM algorithm using iterative maps showed the best performance. DCS-ELM algorithm using chebyshev and logistic maps follow DCS-ELM using iterative maps.

**Figure 5:**
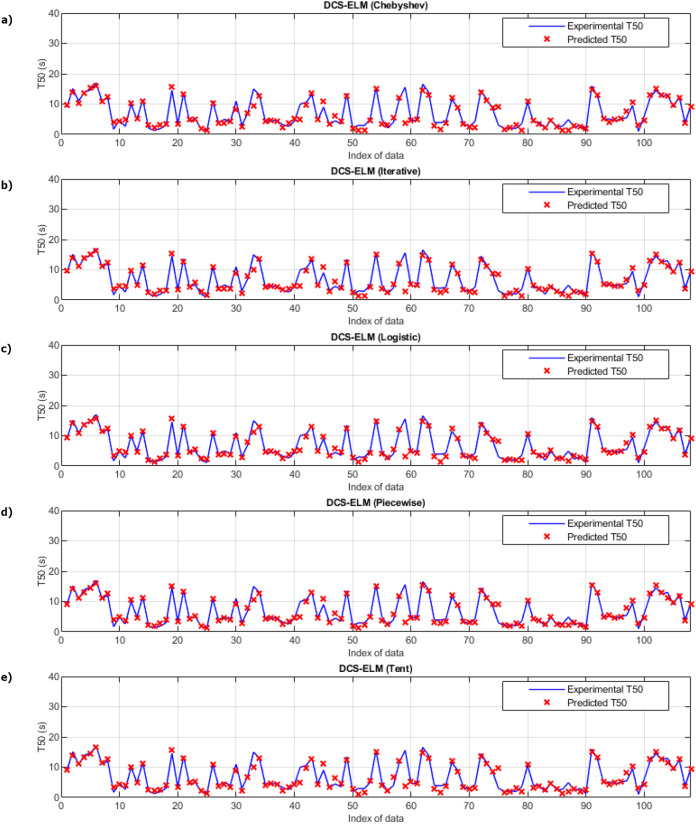
Experimental and predictive values of DCS-ELM algorithm in T50 experiment. (A) Chebyshev. (B) Iterative. (C) Logistic. (D) Piecewise. (E) Tent.

**Figure 6:**
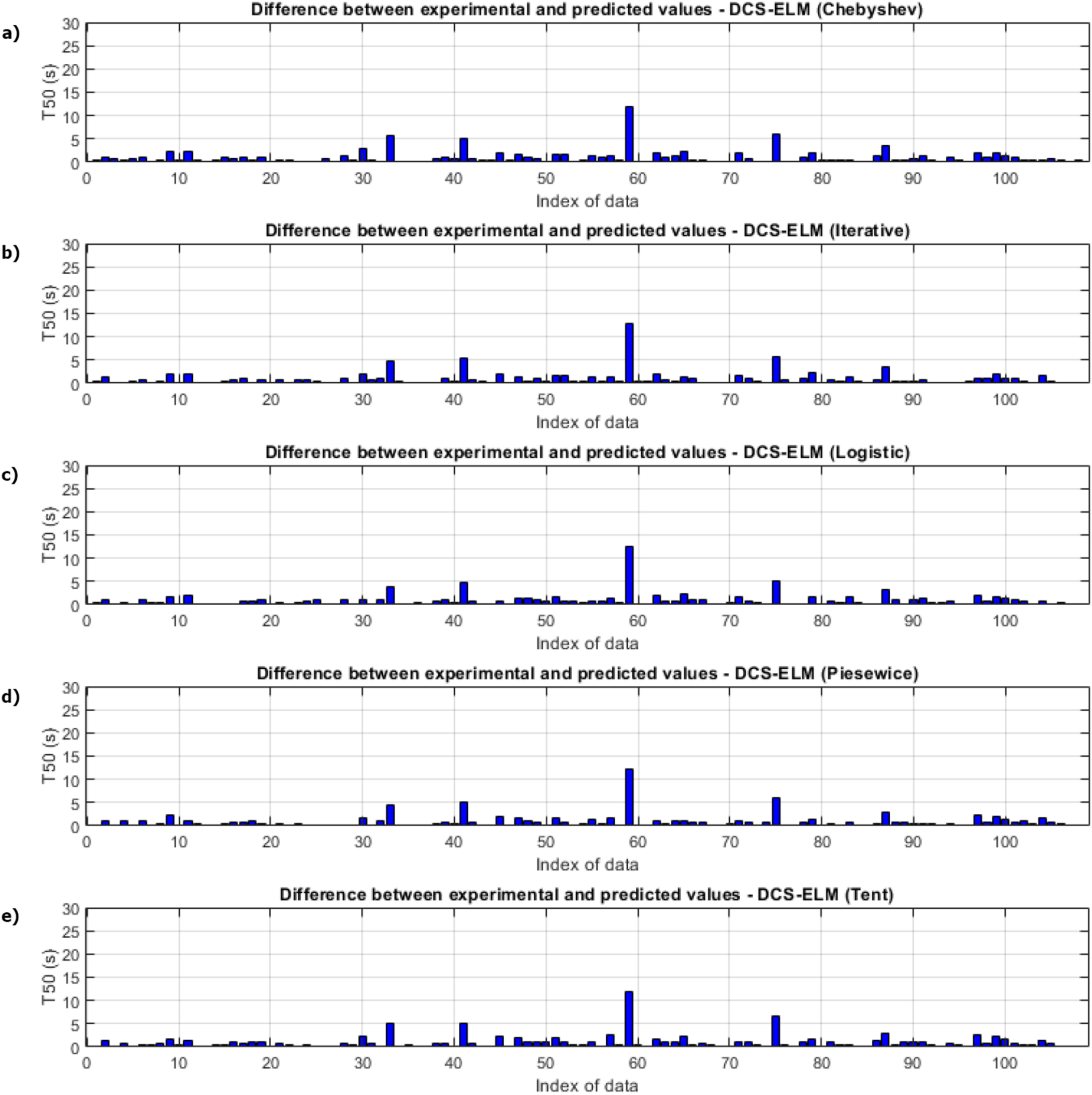
Differences between the experimental and predictive values of DCS-ELM algorithm in the T50 experiment. (A) Chebyshev. (B) Iterative. (C) Logistic. (D) Piecewise. (E) Tent.

Figure 5 shows the ELM algorithm of the T50 experiment and the prediction and experimental values of 5 different DCS-ELM algorithms. Figure 6 shows the differences between prediction and experimental values. As seen in Figs. 5 and 6, the algorithms have shown similar performances to each other. Logistic map-based DCS-ELM algorithm has succeeded in producing the best predictive values. The piecewise map based DCS-ELM algorithm has performed very close to the logistic map based DCS-ELM algorithm.

**Figure 7:**
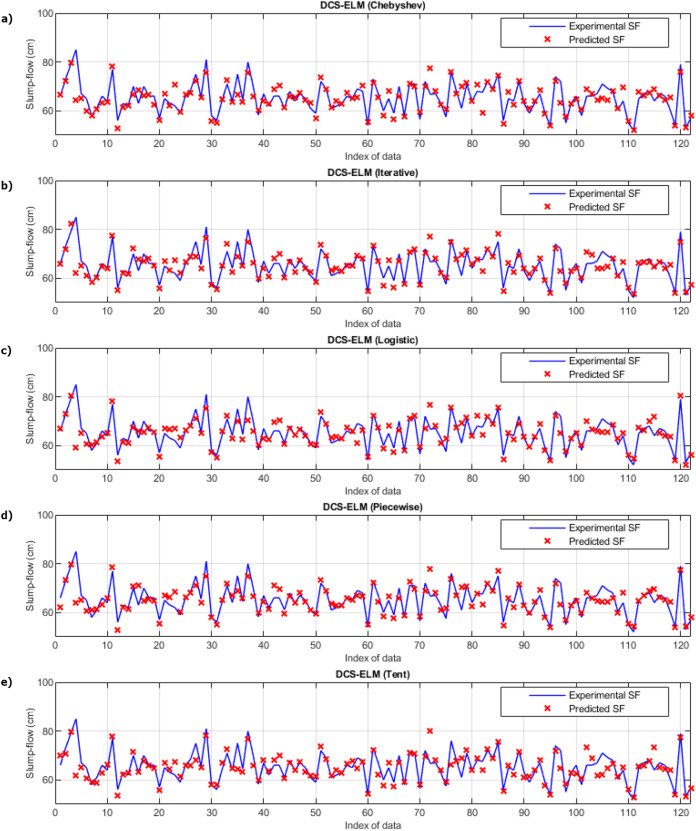
Experimental and predictive values of DCS-ELM algorithm in the slump-flow experiment. (A) Chebyshev. (B) Iterative. (C) Logistic. (D) Piecewise. (E) Tent.

**Figure 8:**
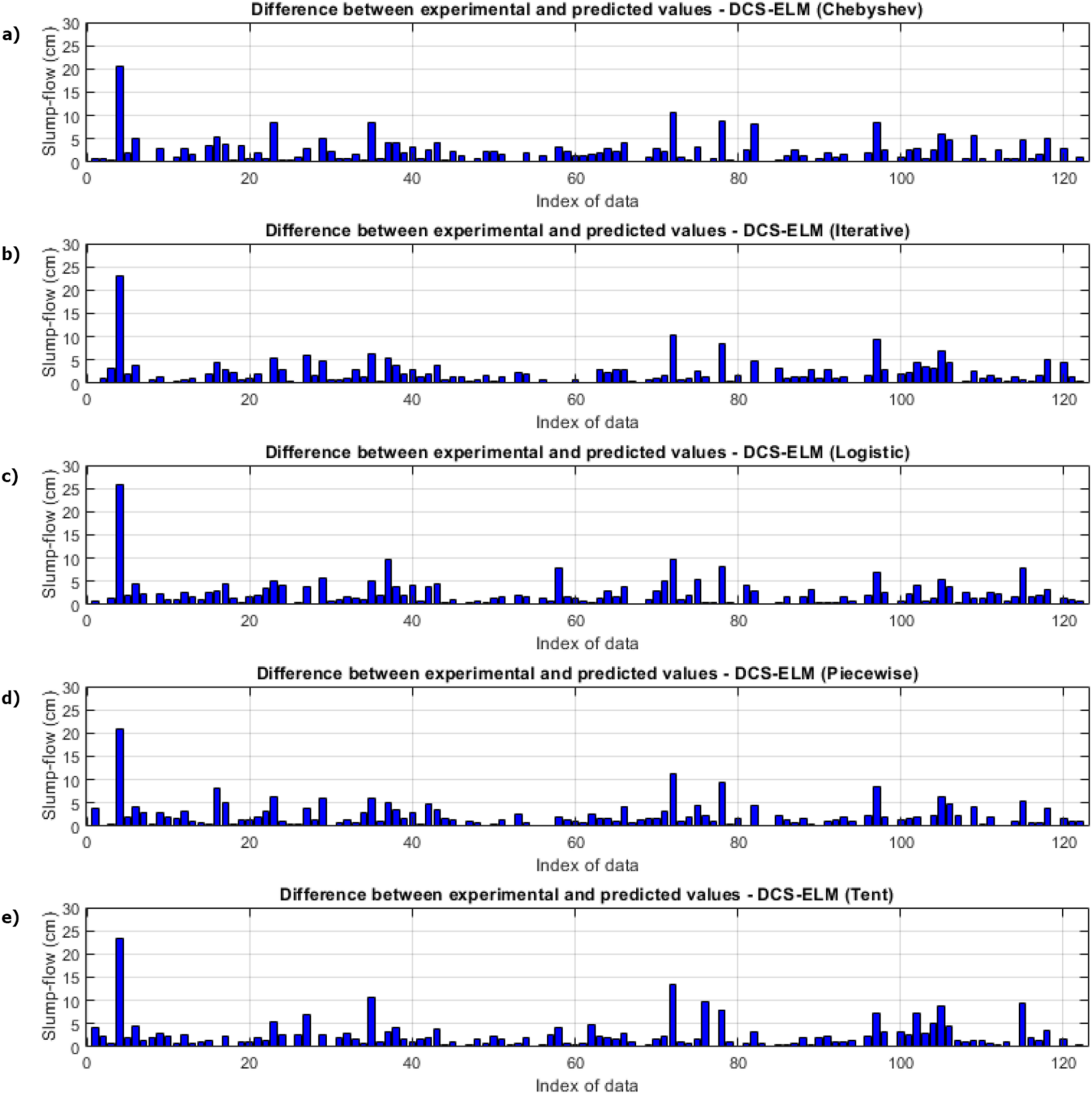
Differences between the experimental and predictive values of DCS-ELM algorithm in the slump-flow experiment. (A) Chebyshev. (B) Iterative. (C) Logistic. (D) Piecewise. (E) Tent.

In Fig. 7, the ELM algorithm of the slump-flow experiment and the prediction and experimental values of 5 different DCS-ELM algorithms are given and the differences between the prediction and experimental values are shown in Fig. 8. As can be seen from Figs. 7 and 8, the most successful performance in the slump-flow experiment was shown by the DCS-ELM algorithm using iterative map. The DCS-ELM algorithm using piecewise map produced predictive values close to the DCS-ELM algorithm using iterative map. Tent and logistic map-based DCS-ELM algorithm produced more distant values in predicted values than expected experimental values.

**Figure 9:**
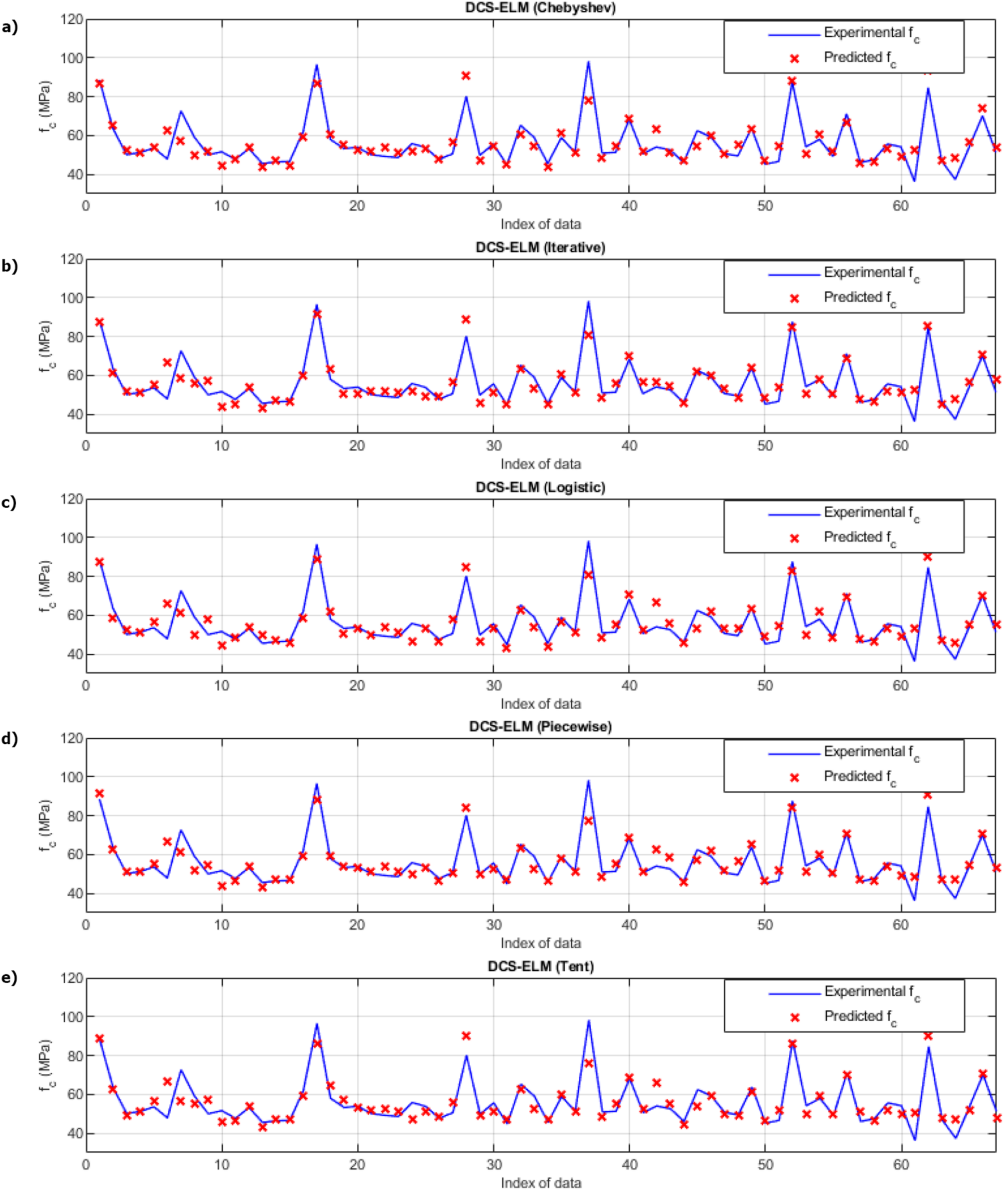
Experimental and predictive values of DCS-ELM algorithm in compressive strength test. (A) Chebyshev. (B) Iterative. (C) Logistic. (D) Piecewise. (E) Tent.

**Figure 10:**
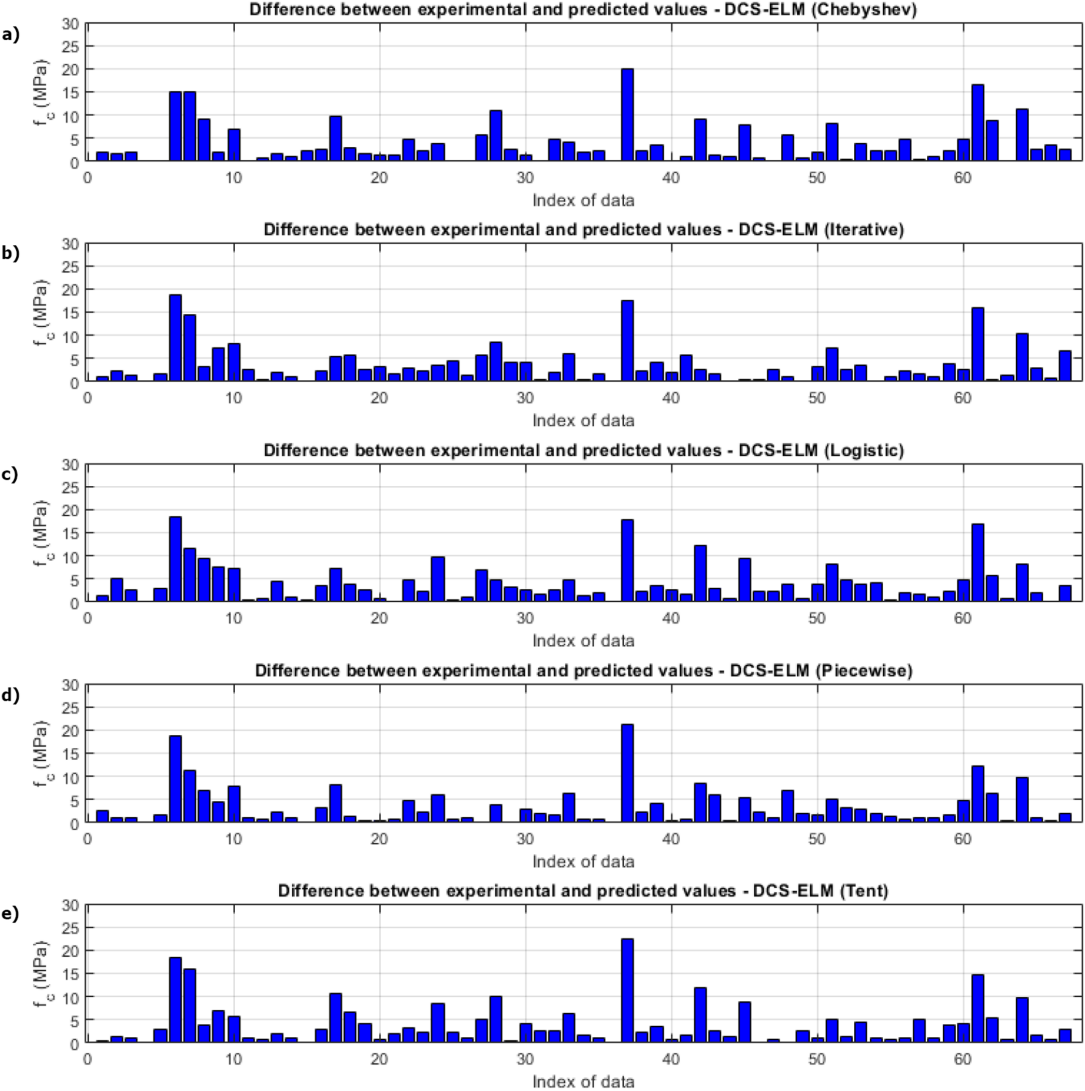
Differences between the experimental and predicted values of DCS-ELM algorithm in compressive strength test. (A) Chebyshev. (B) Iterative. (C) Logistic. (D) Piecewise. (E) Tent.

Figure 9 shows the ELM algorithm of the compressive strength test and the prediction and experimental values of 5 different DCS-ELM algorithms. Figure 10 shows the differences between prediction and experimental values. As can be seen from Figs. 9 and 10, the methods in the compressive strength test have produced predictive values that are not far from each other. The DCS-ELM algorithm, which uses piecewise map, has managed to produce relatively better predictive values.

R^2^, RMSE and MAE values for basic ELM and DCS-ELM are given separately in Table 12. In the V-funnel experiment, ELM algorithm obtained values of 0.6617, 2.8365 and 2.0797 for R^2^, RMSE and MAE values, respectively. Chebyshev map based DCS-ELM algorithm obtained values of 0.6894, 2.4968 and 2.2025 for R^2^, RMSE and MAE values, respectively. Iterative map-based DCS-ELM algorithm obtained values of 0.7056, 2.0798 and 1.6262 for R^2^, RMSE and MAE values, respectively. Logistic map based DCS-ELM algorithm obtained values of 0.6949, 2.8948 and 2.4825 for R^2^, RMSE and MAE values, respectively. The piecewise map based DCS-ELM algorithm obtained values of 0.6794, 2.7398 and 2.1089 for R^2^, RMSE and MAE values, respectively. Tent map-based DCS-ELM algorithm obtained values of 0.6656, 2.8453 and 2.5755 for R^2^, RMSE and MAE values, respectively.

**Table 12:** Comparison of the performances of ELM and DCS-ELM algorithms in SFRSCC data sets.

Data sets	Evaluation metrics	ELM (*k* = 100)	DCS-ELM (Chebyshev)	DCS-ELM (Iterative)	DCS-ELM (Logistic)	DCS-ELM (Piecewise)	DCS-ELM (Tent)
V-funnel	*R*^2^	0.6617	0.6894	0.7056	0.6949	0.6794	0.6656
	RMSE	2.8365	2.4968	2.0798	2.8948	2.7398	2.8453
	MAE	2.0797	2.2025	1.6262	2.4825	2.1089	2.5755
T50	*R*^2^	0.8565	0.8499	0.8533	0.8687	0.8665	0.8528
	RMSE	1.0099	0.9122	1.0020	0.8541	1.0644	1.0727
	MAE	0.7960	0.7198	0.7318	0.6539	0.8750	0.7789
Slump-flow	*R*^2^	0.6324	0.6862	0.6999	0.6453	0.6942	0.6251
	RMSE	2.7543	2.4183	2.1685	2.8308	2.1747	3.0299
	MAE	1.9247	1.7383	1.5668	2.0878	1.4862	1.7589
fc	*R*^2^	0.7604	0.7905	0.8183	0.7882	0.8235	0.7750
	RMSE	5.5749	6.2847	5.2188	4.4419	4.8539	4.8080
	MAE	4.1536	4.8892	3.7992	3.3678	3.3575	3.5935

In the T50 experiment, ELM algorithm obtained values of 0.8565, 1.0099 and 0.7960 for R^2^, RMSE and MAE values, respectively. Chebyshev map-based DCS-ELM algorithm obtained values of 0.8499, 0.9122 and 0.7198 for R^2^, RMSE and MAE values, respectively. The iterative map-based DCS-ELM algorithm obtained the values of 0.8533, 1.0020 and 0.7318 for R^2^, RMSE and MAE values, respectively. Logistic map-based DCS-ELM algorithm obtained values of 0.8687, 0.8541 and 0.6539 for R^2^, RMSE and MAE values, respectively. The piecewise map based DCS-ELM algorithm obtained values of 0.8665, 1.0644 and 0.8750 for R^2^, RMSE and MAE values, respectively. Tent map based DCS-ELM algorithm obtained values of 0.8528, 1.0727 and 0.7789 for R^2^, RMSE and MAE values, respectively.

In the slump-flow experiment, the ELM algorithm obtained values of 0.6324, 2.7543 and 1.9247 for R^2^, RMSE and MAE values, respectively. Chebyshev map-based DCS-ELM algorithm obtained values of 0.6862, 2.4183 and 1.7383 for R^2^, RMSE and MAE values, respectively. Iterative map-based DCS-ELM algorithm obtained values of 0.6999, 2.1685 and 1.5668 for R^2^, RMSE and MAE values, respectively. Logistic map based DCS-ELM algorithm obtained values of 0.6453, 2.8308 and 2.0878 for R^2^, RMSE and MAE values, respectively. The piecewise map based DCS-ELM algorithm obtained values of 0.6942, 2.1747 and 1.4862 for R^2^, RMSE and MAE values, respectively. Tent map-based DCS-ELM algorithm obtained values of 0.6251, 3.0299 and 1.7589 for R^2^, RMSE and MAE values, respectively.

In the compressive strength experiment, ELM algorithm obtained values of 0.7604, 5.5749 and 4.1536 for R^2^, RMSE and MAE values, respectively. Chebyshev map-based DCS-ELM algorithm obtained values of 0.7905, 6.2847 and 4.8892 for R^2^, RMSE and MAE values, respectively. The iterative map based DCS-ELM algorithm obtained values of 0.8183, 5.2188 and 3.7992 for R^2^, RMSE and MAE values, respectively. Logistics map based DCS-ELM algorithm obtained values of 0.7882, 4.4419 and 3.3678 for R^2^, RMSE and MAE values, respectively. The piecewise map based DCS-ELM algorithm obtained values of 0.8235, 4.8539 and 3.3575 for R^2^, RMSE and MAE values, respectively. Tent map based DCS-ELM algorithm obtained values of 0.7750, 4.8080 and 3.5935 for R^2^, RMSE and MAE values, respectively.

**Figure 11:**
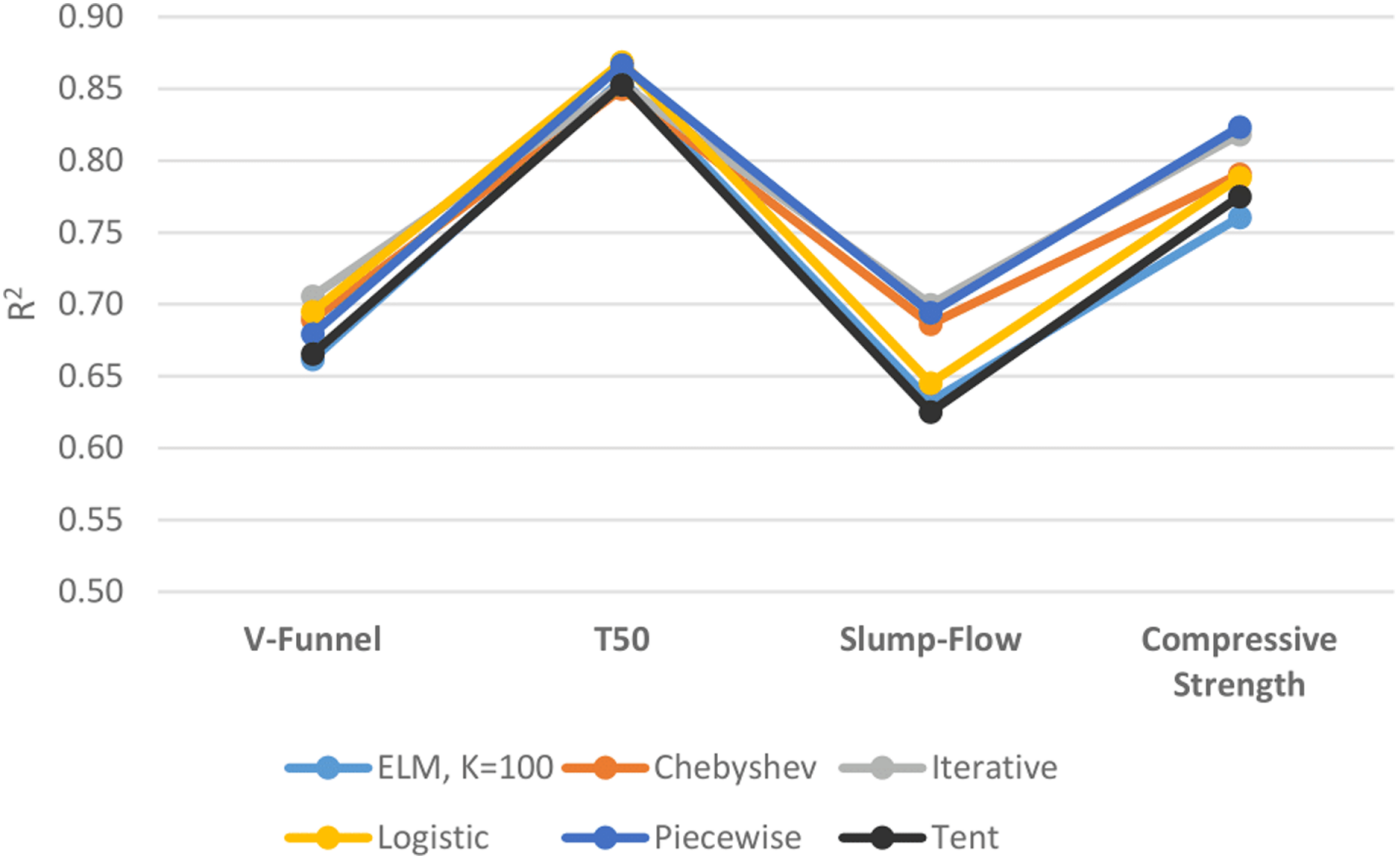
Comparison of ELM and DCS-ELM algorithms according to *R*^2^ value in experiments.

Figure 11 shows the performances of ELM and DCS-ELM algorithms in 4 different data sets according to the R^2^ value. In the V-Funnel experiment, it is an iterative map-based DCS-ELM algorithm that gives the best result according to the R^2^ evaluation criteria. This algorithm performed 6.63% better than the basic ELM algorithm, 6% better than the tent map based DCS-ELM algorithm, 3.86% better than the piecewise map based DCS-ELM algorithm, 2.35% better than the Chebyshev map based DCS-ELM algorithm and 1.54% better than the logistic map based DCS-ELM algorithm.

In the T50 experiment, it is the logistic map based DCS-ELM algorithm that gives the best result according to the R^2^ evaluation criteria. This algorithm has performed 2.21% better than Chebyshev map based DCS-ELM algorithm, 1.86% better than the tent map based DCS-ELM algorithm, 1.80% better than the iterative map based DCS-ELM algorithm, 1.42% better than the basic ELM algorithm and 0.25% better than the piecewise map based DCS-ELM algorithm.

In the slump-flow experiment it is the iterative map based DCS-ELM algorithm that gives the best result according to the R^2^ evaluation criteria. This algorithm has performed 11.97% better than the tent map-based DCS-ELM algorithm, 10.67% better than the basic ELM algorithm, 8.46% better than the logistic map-based DCS-ELM algorithm, 2% better than the Chebyshev map-based DCS-ELM algorithm and 0.82% better than the piecewise map-based DCS-ELM algorithm.

In the compressive strength experiment it is the iterative map based DCS-ELM algorithm that gives the best result according to the R^2^ evaluation criteria. This algorithm has performed 8.3% better than the basic ELM algorithm, 6.26% better than the tent map based DCS-ELM algorithm, 4.48% better than the logistic map based DCS-ELM algorithm, 4.17% better than Chebyshev map based DCS-ELM and 0.64% better than the iterative map based DCS-ELM algorithm.

**Figure 12:**
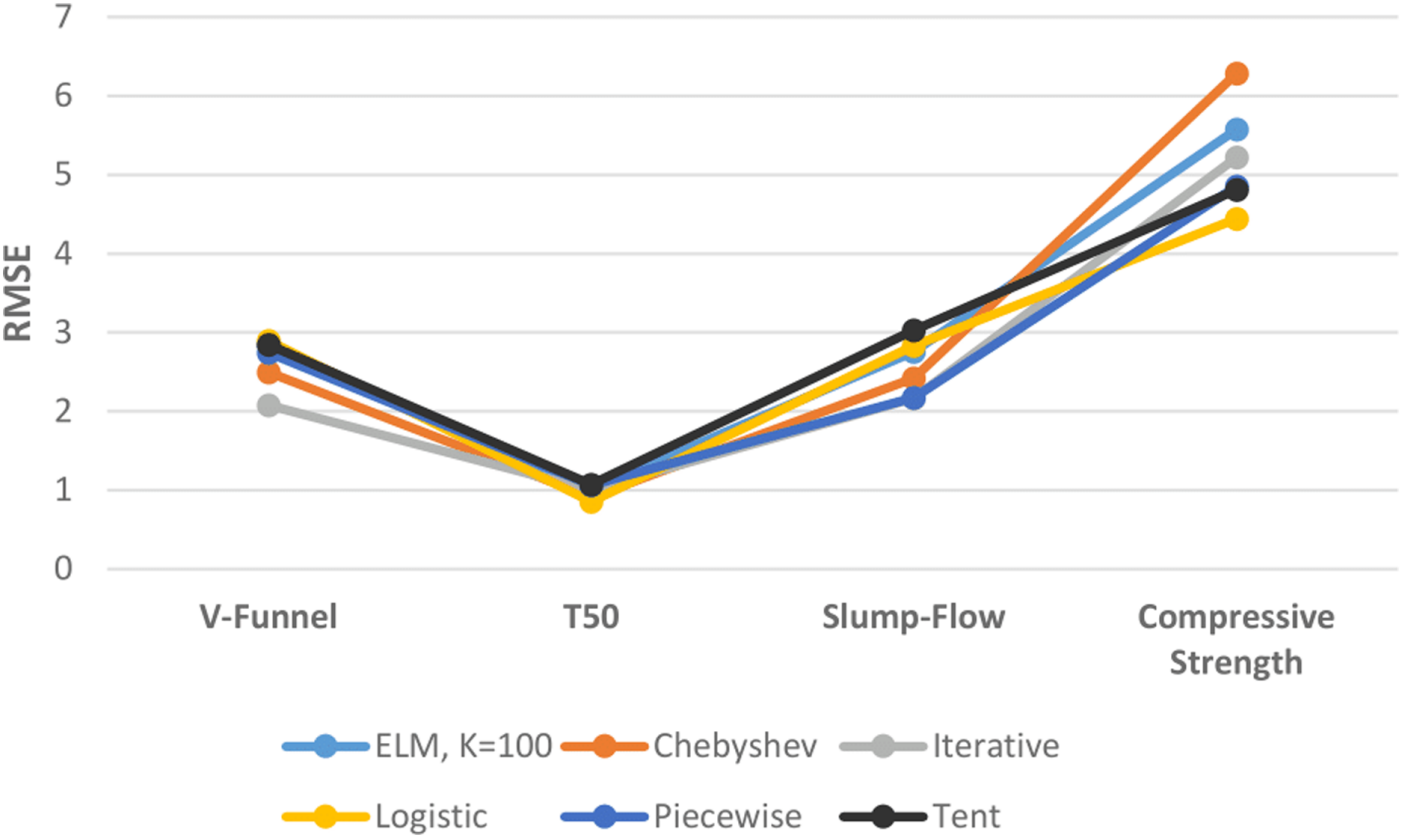
Comparison of ELM and DCS-ELM algorithms according to RMSE value in experiments.

Figure 12 shows the performances of ELM and DCS-ELM algorithms in 4 different data sets according to the RMSE value. In the V-Funnel experiment, the iterative map-based DCS-ELM algorithm, which gives the best performance according to the RMSE evaluation criteria, is 28.15% better than the logistic map-based DCS-ELM algorithm, 26.9% better than the tent map-based DCS-ELM algorithm, 26.68% better than the basic ELM algorithm, 24.09% better than the piecewise map-based DCS-ELM algorithm and 16.7% better than the Chebyshev map-based DCS-ELM algorithm.

In the T50 experiment, the logistic map-based DCS-ELM algorithm, which gives the best performance according to the RMSE evaluation criteria, is 20.38% better than the tent map-based DCS-ELM algorithm, 19.76% better than the piecewise map-based DCS-ELM algorithm, 15.43% better than the basic ELM algorithm, 14.76% better than the iterative map-based DCS-ELM algorithm and 6.37% better than the Chebyshev map-based DCS-ELM algorithm.

In the slump-flow experiment, the iterative map-based DCS-ELM algorithm, which gives the best performance according to the RMSE evaluation criteria, is 28.43% better than the tent map-based DCS-ELM algorithm, 23.4% better than the logistic map-based DCS-ELM algorithm, 21.27% better than the basic ELM algorithm, 10.33% better than the Chebyshev map-based DCS-ELM algorithm and 0.29% better than the piecewise map-based DCS-ELM algorithm.

In the compressive strength experiment, the logistic map-based DCS-ELM algorithm, which gives the best performance according to the RMSE evaluation criteria, is 29.32% better than the tent map-based DCS-ELM algorithm, 20.32% better than the basic ELM algorithm, 14.89% better than the iterative map-based DCS-ELM algorithm, 8.49% better than the piecewise map-based DCS-ELM algorithm and 6.37% better than the Chebyshev map-based DCS-ELM algorithm.

**Figure 13:**
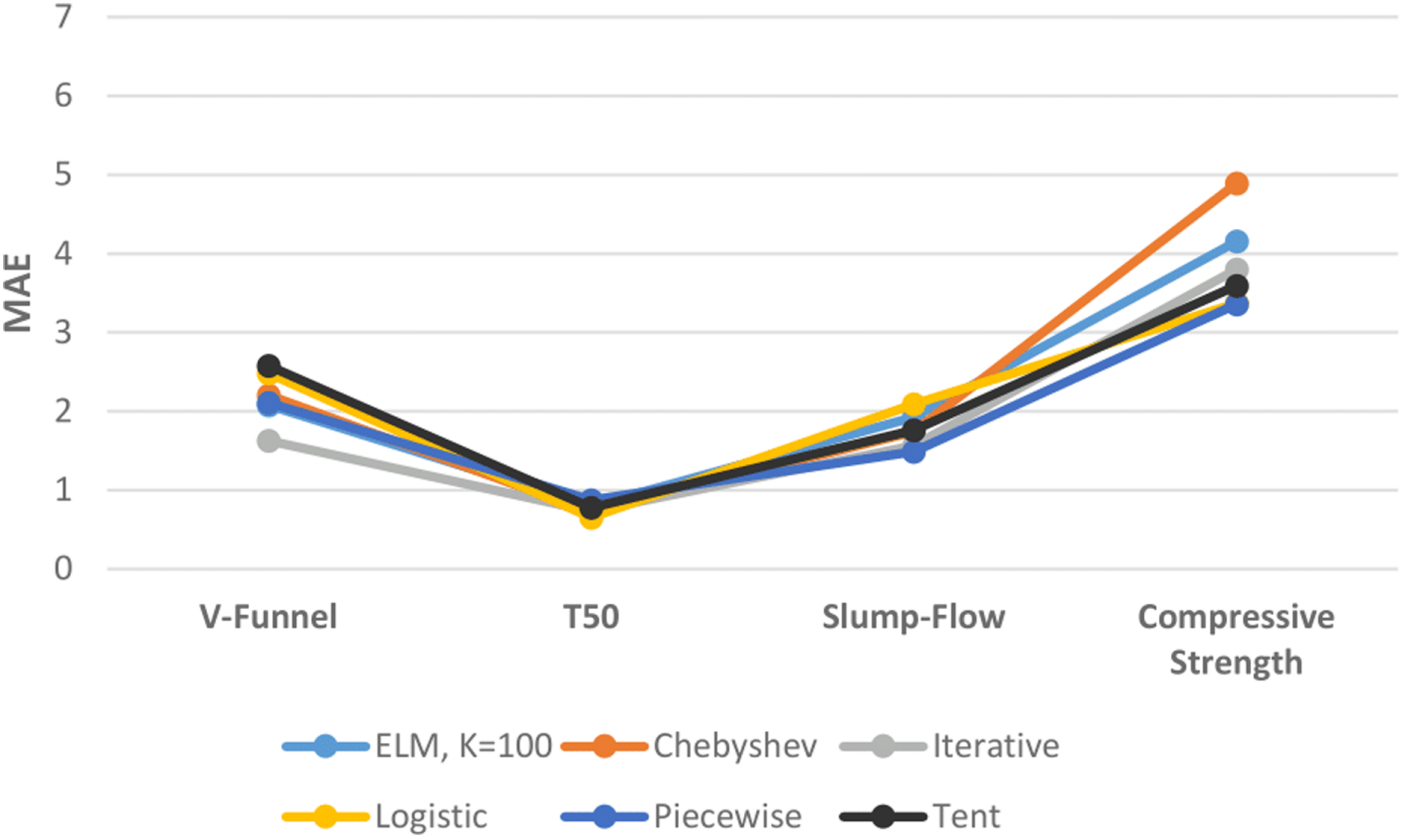
Comparison of ELM and DCS-ELM algorithms according to the MAE value in experiments.

Figure 13 shows the performances of ELM and DCS-ELM algorithms in 4 different data sets according to the MAE value. In the V-Funnel experiment, the iterative map-based DCS-ELM algorithm, which gives the best performance according to the MAE evaluation criteria, is 36.86% better than the tent map-based DCS-ELM algorithm, 34.49% better than the logistic map-based DCS-ELM algorithm, 26.17% better than the Chebyshev map-based DCS-ELM, 22.89% better than the piecewise map-based DCS-ELM algorithm and 21.81% better than the basic ELM algorithm.

In the T50 experiment, the logistic map-based DCS-ELM algorithm, which gives the best performance according to the MAE evaluation criteria, is 25.27% better than the piecewise map-based DCS-ELM algorithm, 17.87% better than the basic ELM algorithm, 16.04% better than the tent map-based DCS-ELM, 10.65% better than the iterative map-based DCS-ELM algorithm and 9.16% better than the Chebyshev map-based DCS-ELM algorithm.

In the slump-flow experiment, the iterative map-based DCS-ELM algorithm, which gives the best performance according to the MAE evaluation criteria, is 24.95% better than the logistic map-based DCS-ELM algorithm, 18.6% better than the basic ELM algorithm, 10.92% better than the tent map-based DCS-ELM, 9.87% better than the Chebyshev map-based DCS-ELM algorithm and 5.42% better than the piecewise map-based DCS-ELM algorithm.

In the compressive strength experiment, the piecewise map-based DCS-ELM algorithm, which gives the best performance according to the MAE evaluation criteria, is 31.32% better than the Chebyshev map-based DCS-ELM algorithm, 19.17% better than the basic ELM algorithm, 11.63% better than the iterative map-based DCS-ELM, 6.57% better than the tent map-based DCS-ELM algorithm and 0.31% better than the logistic map-based DCS-ELM algorithm. It has been demonstrated that the DCS-ELM algorithm produces better results than the ELM algorithm in all SFRSCC data sets.

### General comparison of all data sets

As a result of the study, it was observed that the use of chaotic maps in the ELM algorithm increased the success performance in the SFRSCC and public data sets. However, there is no clear superiority between five different maps. The performance rankings of chaotic maps vary according to the evaluation criteria and the type of data set. When it will be adapted to different data sets, it is recommended to determine the chaotic map by trial and error method. The results of all methods and data sets used in the article are given in Table 13. Table 14 shows the success rankings of the algorithms used in 8 different data sets. When the average values were taken according to 8 different data sets, it was seen that the iterative chaotic map based DCS-ELM method achieved the best average. Piecewise map-based DCS-ELM method took the second place. It has been observed that DCS-ELM gives better results than LR, SVR, WELM and KELM algorithms. It has been observed that the DCS-ELM method gives a much better performance as a percentage, especially in data sets where the ELM method has a low performance rate.

**Table 13:** Results of all datasets.

	EM	LR	SVR	WELM *k* = 100	KELM *k* = 100	ELM *k* = 100	DCS-ELM Chebyshev	DCS-ELM Iterative	DCS-ELM Logistic	DCS-ELM Piecewise	DCS-ELM Tent
Energy1	*R*^2^	0.92	0.91	0.9336	0.9055	0.9809	0.8672	0.9905	0.8723	0.9826	0.9794
	RMSE	2.9421	2.09736	2.5596	3.0598	1.5470	5.5700	1.2450	4.9494	1.6421	1.2402
	MAE	2.0877	2.0435	1.9333	2.3510	1.0524	3.1354	0.8039	2.8124	1.0862	0.8969
Energy2	*R*^2^	0.89	0.88	0.9516	0.8838	0.9730	0.9478	0.9779	0.9626	0.9708	0.9636
	RMSE	3.2188	3.2484	2.0968	3.1851	2.0885	2.2023	1.7219	2.4084	1.9607	2.6307
	MAE	2.2643	2.2441	1.5415	2.3735	1.1709	1.5281	1.0744	1.5189	1.1168	1.2881
House	*R*^2^	0.57	0.56	0.6041	0	0.5677	0.5932	0.6087	0.5676	0.6143	0.5292
	RMSE	8.9474	9.041	8.0768	29.2569	7.3118	7.2347	6.6873	7.4086	6.5335	7.2982
	MAE	6.2745	6.2238	5.7113	24.6785	5.5837	5.7505	5.0731	5.7930	5.2752	5.7343
Servo	*R*^2^	0.48	0.17	0.4936	0.6997	0.7893	0.7189	0.8271	0.2270	0.8265	0.7924
	RMSE	1.1348	1.4304	1.0887	0.7698	0.4511	0.5118	0.3574	0.8743	0.6183	0.3832
	MAE	0.9169	0.7850	0.7978	0.5153	0.3549	0.4323	0.2626	0.6280	0.4905	0.2798
Vfunnel	*R*^2^	0.7820	0.8011	0.6995	0.6746	0.6617	0.6894	0.7056	0.6949	0.6794	0.6656
	RMSE	2.2727	2.1707	2.4589	2.6789	2.8365	2.4968	2.0798	2.8948	2.7398	2.8453
	MAE	1.8330	1.7657	1.9675	2.2002	2.0797	2.2025	1.6262	2.4825	2.1089	2.5755
T50	*R*^2^	0.7669	0.7705	0.8569	0.7577	0.8565	0.8499	0.8533	0.8687	0.8665	0.8528
	RMSE	2.2360	2.2190	1.5121	2.1675	1.0099	0.9122	1.0020	0.8541	1.0644	1.0727
	MAE	1.6917	1.5372	0.9690	1.6974	0.7960	0.7198	0.7318	0.6539	0.8750	0.7789
Slump-flow	*R*^2^	0.6123	0.6607	0.6247	0.4873	0.6324	0.6862	0.6999	0.6453	0.6942	0.6251
	RMSE	3.8928	3.6417	3.3328	4.4134	2.7543	2.4183	2.1685	2.8308	2.1747	3.0299
	MAE	2.9481	2.6285	2.3177	3.3497	1.9247	1.7383	1.5668	2.0878	1.4862	1.7589
fc	*R*^2^	0.7307	0.7630	0.7541	0.7298	0.7604	0.7905	0.8183	0.7882	0.8235	0.7750
	RMSE	6.5560	6.1514	5.1207	5.9819	5.5749	6.2847	5.2188	4.4419	4.8539	4.8080
	MAE	4.6861	4.3750	3.8332	4.5412	4.1536	4.8892	3.7992	3.3678	3.3575	3.5935

**Table 14:** Ranking of algorithms according to RMSE value.

	LR	SVR	WELM *k* = 100	KELM *k* = 100	ELM *k* = 100	DCS-ELM Chebyshev	DCS-ELM Iterative	DCS-ELM Logistic	DCS-ELM Piecewise	DCS-ELM Tent
Energy1	7	5	6	8	3	10	2	9	4	1
Energy2	9	10	4	8	3	5	1	6	2	7
House	8	9	7	10	5	3	2	6	1	4
Servo	9	10	8	6	3	4	1	7	5	2
Vfunnel	3	2	4	6	8	5	1	10	7	9
T50	10	9	7	8	4	2	3	1	5	6
Slump-flow	9	8	7	10	4	3	1	5	2	6
fc	10	8	4	7	6	9	5	1	3	2
Mean rank	8.13	7.63	5.88	7.88	4.50	5.13	2.00	5.63	3.63	4.63

## Conclusions

In this study, a novel method named DCS-ELM is proposed to improve the ELM algorithm. In this proposed method, 5 different chaotic maps are used. These chaotic maps are Chebyshev map, iterative map, logistic map, piecewise map and tent map. It has been shown that the performance of the DCS-ELM algorithm changes according to the chaotic map used. The DCS-ELM method proposed in this study has been tested in 8 different data sets. The common parameters of the models designed in each data set are used the same. In addition, the test and training data sets used during the testing of the models were used the same. As a result of the study, it was observed that the DCS-ELM algorithm is more stable, problem solving ability is more generalized and higher performance thanks to the use of chaotic maps in the ELM algorithm. Especially in datasets where ELM or other algorithms showed poor performance, DCS-ELM algorithm was able to perform better than basic ELM, KELM, WELM, LR and SVR. It has been shown that problems such as accumulating randomly assigned number values in a certain place and repeating numbers can be prevented by using chaotic maps. The DCS-ELM algorithm is provided to reach the best performance faster. The proposed discrete-time chaotic systems extreme learning machine algorithm can be appropriately used in regression problems. Novel discrete time chaotic systems based machine learning algorithm can be effectively used in different complex datasets. These proposed methods are novel and more detailed work can be done with parallel or distributed application. In addition, different studies can be done by adapting the chaotic maps to different versions of the ELM algorithm.

## Supplemental Information

10.7717/peerj-cs.411/supp-1 Supplemental Information 1 DCS-ELM Code.

10.7717/peerj-cs.411/supp-2 Supplemental Information 2 Data Description.

10.7717/peerj-cs.411/supp-3 Supplemental Information 3 Public Dataset Description.

10.7717/peerj-cs.411/supp-4 Supplemental Information 4 V-funnel Dataset.

10.7717/peerj-cs.411/supp-5 Supplemental Information 5 T50 Dataset.

10.7717/peerj-cs.411/supp-6 Supplemental Information 6 Slump-flow Dataset.

10.7717/peerj-cs.411/supp-7 Supplemental Information 7 Fc Dataset.

## Additional Information and Declarations

### Data Availability

The following information was supplied regarding data availability: Datasets and codes are available at GitHub: https://github.com/mustafaulas/DCS-ELM. Data is also available at the UCI Machine Learning Repository: - House dataset: https://archive.ics.uci.edu/ml/datasets/Real+estate+valuation+data+set. - Servo Dataset: https://archive.ics.uci.edu/ml/datasets/Servo. - Energy Dataset: https://archive.ics.uci.edu/ml/datasets/Energy+efficiency#.
